# Reduced gonadotroph stimulation by ethanolamine plasmalogens in old bovine brains

**DOI:** 10.1038/s41598-021-84306-6

**Published:** 2021-02-26

**Authors:** Hiroya Kadokawa, Miyako Kotaniguchi, Onalenna Kereilwe, Shinichi Kitamura

**Affiliations:** 1 Faculty of Veterinary Medicine, Yamaguchi University, Yamaguchi-shi, Yamaguchi-ken 1677-1 Japan; 2 Centre for Research and Development of Bioresources, Osaka Prefecture University, 1-2 Gakuen-cho, Naka-ku, Sakai, 599-8531 Japan

**Keywords:** Reproductive disorders, Animal physiology

## Abstract

Ethanolamine plasmalogens (EPls), unique alkenylacyl-glycerophospholipids, are the only known ligands of G-protein-coupled receptor 61—a novel receptor co-localised with gonadotropin-releasing hormone receptors on anterior pituitary gonadotrophs. Brain EPl decreases with age. Commercial EPl—extracted from the cattle brain (unidentified age)—can independently stimulate FSH secretion from gonadotrophs. We hypothesised that there exists an age-related difference in the quality, quantity, and ability of bovine brain EPls to stimulate bovine gonadotrophs. We compared the brains of young (about 26 month old heifers) and old (about 90 month old cows) Japanese Black bovines, including EPls obtained from both groups. Additionally, mRNA expressions of the EPl biosynthesis enzymes, glyceronephosphate O-acyltransferase, alkylglycerone phosphate synthase, and fatty acyl-CoA reductase 1 (FAR1) were evaluated in young and old hypothalami. The old-brain EPl did not stimulate FSH secretion from gonadotrophs, unlike the young-brain EPl. Molecular species of EPl were compared using two-dimensional liquid chromatography-mass spectrometry. We identified 20 EPl molecular species of which three and three exhibited lower (*P* < 0.05) and higher (*P* < 0.05) ratios, respectively, in old compared to young brains. In addition, quantitative reverse transcription-polymerase chain reaction detected higher FAR1 levels in the POA, but not in the ARC&ME tissues, of old cows than that of fertile young heifers. Therefore, old-brain EPl may be associated with age-related infertility.

## Introduction

Reproduction is controlled by the hypothalamus, which contains neurons that secrete gonadotropin-releasing hormone (GnRH). This, in turns, binds to GnRH receptors on the gonadotroph plasma membrane in the anterior pituitary to stimulate the secretion of follicle-stimulating hormone (FSH) and luteinising hormone (LH)^1^. G-protein-coupled receptor 61 (GPR61) is a novel receptor that is co- localise with GnRH receptors on the lipid raft of the gonadotroph surface^2^, with ethanolamine plasmalogen (EPl), a unique alkenylacyl-glycerophospholipid class, being its only known ligands^3^. Even without GnRH, gonadotrophs in heifers were stimulated by cattle extracted commercial EPls to secrete FSH^4^. EPl is decreased in the human brain with ageing, which induces age-related diseases^4,5^. Therefore, EPl may be a molecular link in age-related infertility via GPR61 in gonadotrophs. However, the company from which we purchased EPl did not report the age of the cattle used for extraction. Compared with other organs, the brain contains the highest EPl level^5^. The biosynthesised EPl may be transported from the brain, including the hypothalamus, to gonadotrophs via the systemic circulation or hypophyseal portal system.

EPl contains a fatty alcohol bonded to the glycerol backbone at the sn-1 position with a vinyl-ether bond, and fatty acids bonded to the sn-2 position with an ester bond. We previously suggested the importance of the side chain at the sn-2 position in gonadotroph stimulation^4^. Based on the various possible combinations of fatty alcohol and acids, the brain contains various molecular EPl species. However, there has been no study on differences in the ratios of various EPl molecular species with ageing for two main reasons. First, there had been no appropriate analysis method until we developed a novel two-dimensional liquid chromatography–mass spectrometry (2D LC–MS) system^6^. Second, it is difficult to collect various human brain samples, and the hypothalamic size of laboratory animals is too small for appropriate analysis.

In this study, we used brain samples obtained from cattle. Similar to that of women, cow fertility decreases with age^7^. However, the exact mechanisms underlying this association remain unclear. We hypothesised that there exists an age-related difference in bovine brain EPl quality and quantity, and in its ability to stimulate bovine gonadotrophs. We used EPl extracted from the brain of young and old female bovines with known age and fertility. Glyceronephosphate O-acyltransferase (GNPAT), alkylglycerone phosphate synthase (AGPS), and fatty acyl-CoA reductase 1 (FAR1) are important enzymes involved biosynthesis^5^. Therefore, additionally we compared the mRNA expression of these enzymes in the hypothalamus between young and old female bovines.

## Results

### Weaker gonadotroph stimulation by old-brain EPl

**Figure 1:**
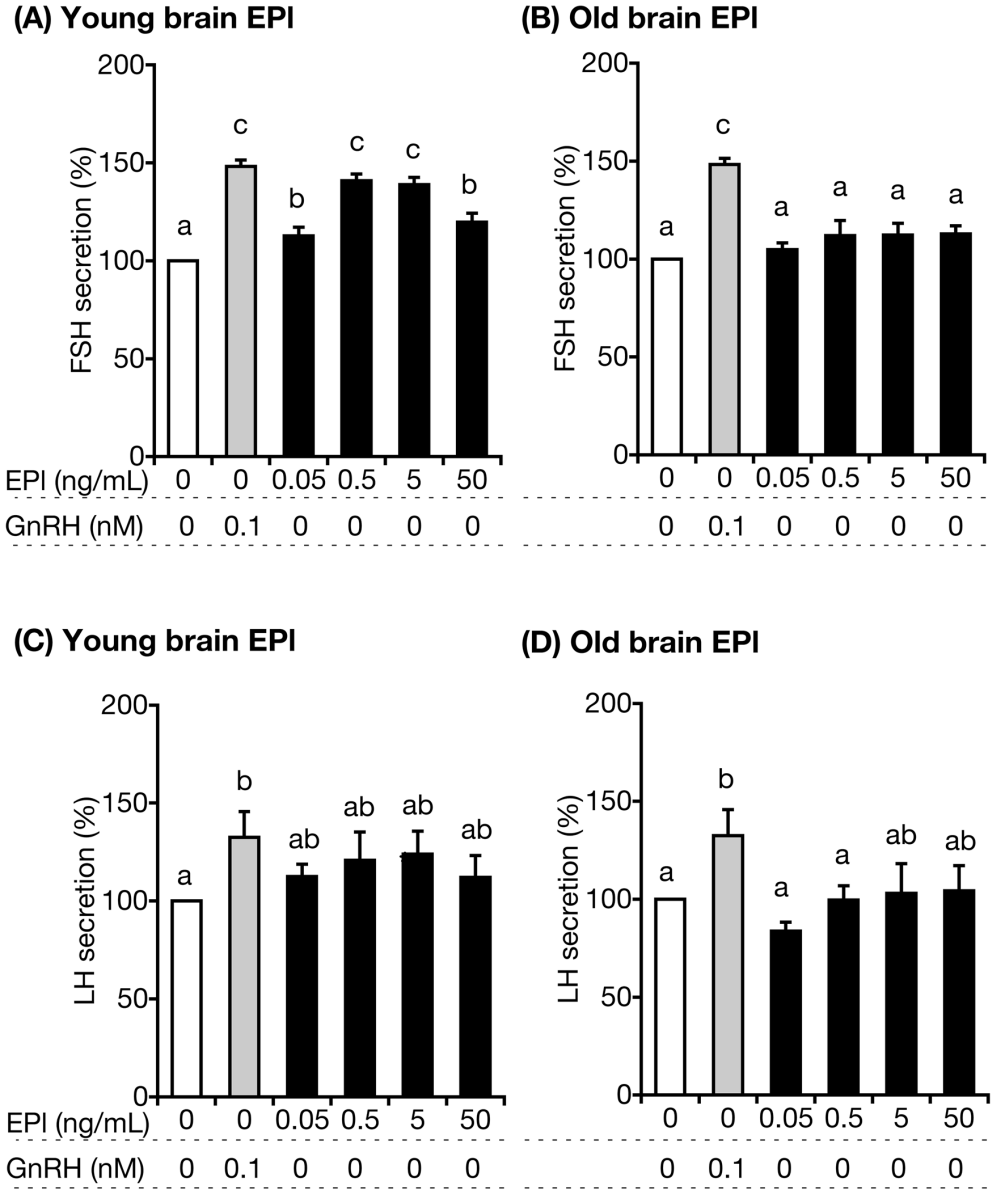
Effects of various concentrations of bovine brain EPl in media lacking GnRH on hormone secretion from cultured AP cells. EPl was obtained from fertile young heifers (**A**, **C**) and old cows (**B**, **D**), and its effect was tested on FSH (**A**, **B**) and LH (**C**, **D**) secretions. FSH and LH concentrations in control cells (cultured in medium lacking EPl and GnRH) were averaged and set as 100%. The mean FSH or LH concentrations in each treatment group were expressed as percentages of the control value. Bars labelled with different letters (a, b, and c) indicate different stimulatory effects (*P* < 0.05; Details of *P* value are presented in Supplementary Tables S1–S4). The bars labelled with the same letter indicate a similar stimulatory effect. Statistical analysis was conducted using one-factor analysis of variance, followed by Fisher’s protected least significant difference test. *AP* anterior pituitary, *EPl* ethanolamine plasmalogen, *GnRH* gonadotropin-releasing hormone, *FSH* follicle-stimulating hormone, *LH* luteinising hormone.

First, we prepared EPl-rich lipids from a whole brain mixture of five fertile young heifers (25.8 ± 0.5 months old), or five old cows (90.8 ± 2.2 months old), following the removal of non-plasmalogen-type phosphatidylethanolamines. Subsequently, we prepared bovine anterior pituitary cells from healthy post-pubertal heifers (25.7 ± 0.4 months old; n = 6) and cultured them for 3.5 days in order to confirm the different anterior pituitary stimulation by young- and old-brain EPl. We incubated the cells with 0, 0.05, 0.5, 5, or 50 ng/mL (final medium concentration) young- or old-brain EPl. The medium samples were harvested 2 h after culture for FSH and LH assays. We observed that all tested concentrations of young brain EPl stimulated FSH (Fig. 1A), but not LH (Fig. 1C) secretion (statistical values are shown in Supplementary Tables S1 and S3 online). The cumulative concentration of FSH secreted from anterior pituitary cells by stimulation of 0.5 ng/mL young-brain EPl over a 2-h period in the absence of GnRH reached the same level as that secreted from cells by GnRH alone. Contrastingly, no old-brain EPl dose stimulated FSH and LH secretion (Fig. 1B,D; statistical values are presented in Supplementary Tables S2 and S4 online).

### Differences in the ratios of EPl molecular species between young and old brains

**Figure 2:**
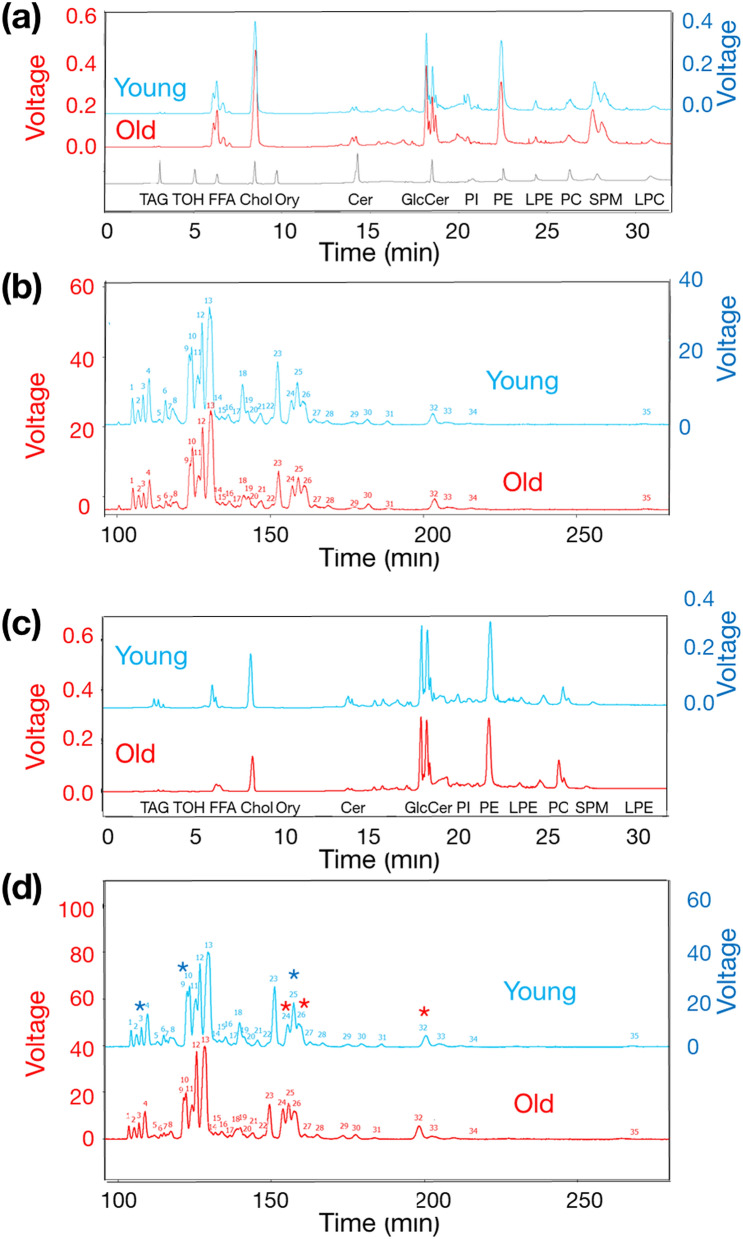
Chromatograms depicting examples of HPLC profiles of bovine EPl. The red font on the left side Y-axis indicates the voltage of the old group, and the blue font on right side Y-axis indicates the voltage of the young group. The chromatograms of young animals were shifted up for clarity, and no difference in the baseline values between young and old animals was observed. (**a**) EPl-rich lipids were extracted from a whole brain mixture obtained from five young heifers and five old cows, and analysed thrice using the 2D LC–MS system. The chromatograms depict an example of a first-dimensional HPLC (normal-phase HPLC and a charged aerosol detector) profile of the extracted EPl-rich lipids and 13 lipid standard compounds (TAG, TOH, FFA, cholesterol, Ory, ceramide, GlcCer, PI, PE, LPE, PC, SPM, and LPC). (**b**) The chromatograms depict an example profile of second-dimensional HPLC separation (reverse-phase-HPLC and a charged aerosol detector) of the El molecular species in the phosphatidylethanolamine fraction, eluted from the first-dimensional HPLC column, exhibiting 35 peaks. (**c**) EPl-rich lipids were extracted from the hypothalami of young heifers (n = 6) and old cows (n = 6) and analysed using the 2D LC–MS system. The chromatograms depict an example profile of the first-dimensional HPLC of hypothalamic EPl-rich lipids, and indicated the elution timing of 13 lipid standard compounds. (**d**) The chromatograms depict an example profile of the second-dimensional HPLC separation of hypothalamic EPl molecular species in the phosphatidylethanolamine fraction, eluted from the first-dimensional HPLC column, from young or old brains, indicating the presence of 35 EPl lipid classes. The peaks refer to the components listed in Table 2. Blue and red asterisks indicate identifiable EPl molecular species that were higher or lower, respectively, in young than in old hypothalami (details are presented in Table 2). *2D LC–MS* two-dimensional liquid chromatography-mass spectrometry, *HPLC* high-performance liquid chromatography, *EPL* ethanolamine plasmalogen, *TAG* tripalmitin, *TOH* D-α-tocopherol, *FFA* palmitic acid, *Ory* cycloartenyl ferulate, *GlcCer* glucosylceramide, *PI* phosphatidylinositol, *PE* 1,2‑Dipalmitoyl‑*sn*‑glycero‑3‑phosphoethanolamine, *LPE* 1‑palmitoyl‑2‑hydroxy‑*sn*‑glycero‑3‑phosphoethanolamine, *PC* 1,2‑dipalmitoyl‑*sn*‑glycero‑3‑phosphocholine, *SPM* sphingomyelin, *LPC* 1‑palmitoyl‑2‑hydroxy‑*sn*‑glycero‑3‑phosphocholine.

We analysed the EPl-rich lipids from the whole brain mixture of five fertile young heifers, or five old cows using the 2D LC–MS system. First-dimensional high-performance liquid chromatography (HPLC) comprised of normal-phase HPLC and a charged aerosol detector. Figure 2a presents an example of a first-dimensional LC profile of EPl-rich lipids in a 0.1-mg mixture of a young- or old whole brain tissue, and 13 lipid standard compounds. We compared the peak area ratio of each lipid class to the total peak area between the young and old brains using a two-tailed unpaired *t*-test (Table 1). The most prevalent major lipid was cholesterol; moreover, there was no difference (*P* = 0.4353) in the ratio of the cholesterol to the total peak area between young and old brains.

**Table 1:** Comparison of the ratio of the peak area of each lipid class to the total peak area of all lipids in young and old brains.

Lipid class	*P* value	Young-brain area (%)^a^		< or > ^b^	Old-brain area (%)^a^	
		Mean	SEM		Mean	SEM
Free fatty acid	0.1012	4.87	0.03		4.97	0.03
Cholesterol	0.4353	18.90	0.06		19.00	0.10
Glucosylceramide	0.0031	10.33	0.03	<	10.93	0.09
Phosphatidylethanolamine containing EPl	0.0001	16.47	0.03	>	13.10	0.10
Lysophosphatidylethanolamine	0.0010	1.70	0.00	>	1.20	0.06
Phosphatidylcholine	0.1277	2.90	0.06		2.47	0.22
Sphingomyelin	0.0014	8.47	0.09	<	9.33	0.07
Lysophosphatidylcholine	0.1848	1.20	0.12		0.93	0.12

*EPl* ethanolamine plasmalogen, *SEM* standard error of the mean.

^a^Ratio of the peak area of each lipid class to the total peak area.

^b^The ratio was higher ( >) or lower ( <) in young than in old brains.

Triplicate analyses were performed for each brain lipid. Statistical analysis was conducted using two-tailed unpaired *t*-tests. A blue or magenta background indicates a lower or higher ratio, respectively, in old than in young hypothalami.

Phosphatidylethanolamines were the second most detected major lipids especially, almost all phosphatidylethanolamines were EPls. The ratio of the phosphatidylethanolamine to the total peak area was lower (*P* = 0.0001) in old than in young brains. Regarding other lipids, the ratio of the lysophosphatidylethanolamine to the total peak area was higher (*P* = 0.001) in young than in old brains. Meanwhile, the ratios of the glucosylceramide (*P* = 0.0031) and sphingomyelin (*P* = 0.0014) peak areas to the total peak area were lower in young than in old brains.

Subsequently, we analysed the EPl molecular species in the phosphatidylethanolamine fraction using second-dimensional, reverse-phase HPLC separation and a charged aerosol detector. Figure 2b presents an example profile of EPl molecular species in the phosphatidylethanolamine fraction, exhibiting 35 peaks. Although we employed a small sample number, there were several differences in the EPl molecular species between the young and old whole brains.

For more precise analyses, we extracted EPl-rich lipids following the removal of non-plasmalogen-type phosphatidylethanolamines from the anterior hypothalamic tissue containing preoptic area (POA tissue), or from the intermediate and posterior hypothalamic tissues containing arcuate nucleus and median eminence (ARC&ME tissues) of fertile young heifers (25.7 ± 0.4 months old; n = 6) and old cows (91.0 ± 1.9 months old; n = 6). We observed differences in the hypothalamic lipid class ratios between the young and old brains. Specifically, the ratio of the phosphatidylethanolamine to the total area was lower (*P* = 0.0001) in old (13.22 ± 0.35%) than in young (15.86 ± 0.25%) brains. We compared the peak area ratio of each EPl molecular species to the total phosphatidylethanolamine area in each hypothalamus between young and old brains using 2D LC–MS. Figure 2c,d present an example profile of first- and second-dimensional HPLC separation. Similar to the whole brain samples, the EPl-rich lipids extracted from the hypothalamus exhibited 35 peaks. The 2D LC–MS system identified 20 EPl molecular species (Table 2). Among them, the ratios of three molecular species [C16:0–C20:4 (the carbon chain at the sn-1 position was C16:0, and the fatty acid at the sn-2 position was C20:4) (*P* = 0.0246), C16:0–C22:4 (*P* = 0.0403), and C18:0-C18:1 (*P* = 0.0048)] were lower in the hypothalami of old than that in the young brains. In contrast, three molecular species [C16:0–C20:1 (*P* = 0.0075), C18:1–C20:1 (*P* = 0.0033), and C18:0–C20:1 (*P* = 0.0039)] exhibited higher ratios in the hypothalamus in old than in young brains.

**Table 2:** Composition and comparison of hypothalamic EPl molecular species extracted from young (n = 6) and old (n = 6) brains.

Peakno	RT (min)	*m/z*^a^	Identified molecular species^b^	*P* value	Young hypothalamus area (%)^d^		< or > ^e^	Old hypothalamus area (%)	
					Mean	SEM		Mean	SEM
1	104.36	748.53	16:0–22:6	0.0774	1.060	0.051		0.922	0.048
2	106.25	774.56	18:1–22:6	0.6005	1.217	0.075		1.157	0.082
3	107.81	724.52	16:0–20:4	0.0246	1.505	0.059	** > **	1.246	0.078
4	109.74	750.54	18:1–20:4	0.3931	3.643	0.178		3.417	0.182
5	112.95	762.60	UID^**c**^	0.3921	0.947	0.075		0.858	0.066
6	114.99	750.56	16:0–22:5	0.1235	0.912	0.109		0.659	0.103
7	116.06	726.55	16:0–20:3	0.8596	0.490	0.030		0.503	0.067
8	117.18	776.55	18:1–22:5	0.4765	2.113	0.135		1.973	0.134
9	118.38	752.54	16:0–22:4	0.0403	5.298	0.189	** > **	4.197	0.428
10	122.57	776.54	18:0–22:6	0.7358	4.725	0.302		4.539	0.442
11	123.42	778.58	18:1–22:4	0.2269	6.543	0.203		5.995	0.375
12	125.43	702.50	16:0–18:1	0.5657	10.065	0.486		9.669	0.456
13	126.78	752.59	18:0–20:4	0.7229	20.015	0.647		19.675	0.671
14	129.31	704.57	UID	0.8043	0.880	0.050		0.896	0.037
15	131.01	728.68	16:0–20:2	0.5544	0.835	0.065		0.888	0.058
16	133.06	766.60	UID	0.1758	1.705	0.102		1.910	0.097
17	135.30	754.20	16:0–22:3	0.2831	0.372	0.036		0.463	0.072
18	137.87	778.58	18:0–22:5	0.2196	2.855	0.284		2.403	0.195
19	139.84	754.62	UID	0.0342	1.305	0.059	** < **	1.530	0.070
20	141.79	742.60	UID	0.0361	0.352	0.034	** < **	0.551	0.075
21	142.34	780.61	UID	0.8247	1.207	0.126		1.239	0.068
22	145.50	806.60	UID	0.1933	0.512	0.042		0.597	0.045
23	149.18	780.56	18:0–22:4	0.0639	7.725	0.324		6.475	0.505
24	151.13	730.57	16:0–20:1	0.0075	3.345	0.149	** < **	4.228	0.219
25	155.43	730.54	18:0–18:1	0.0048	6.453	0.140	** > **	5.845	0.094
26	157.34	756.58	18:1–20:1	0.0033	5.238	0.191	** < **	6.847	0.373
27	158.98	732.60	UID	0.0828	1.203	0.058		1.393	0.079
28	162.62	758.65	UID	0.0103	1.047	0.044	** < **	1.361	0.090
29	166.99	744.69	UID	0.0200	0.880	0.056	** < **	1.170	0.089
30	175.46	782.62	UID	0.0363	0.902	0.066	** < **	1.102	0.050
31	179.73	808.67	UID	0.0010	0.563	0.031	** < **	0.788	0.037
32	186.04	758.60	18:0–20:1	0.0039	2.542	0.113	** < **	3.352	0.186
33	200.85	784.66	UID	0.0039	0.963	0.042	** < **	1.348	0.094
34	204.59	760.60	UID	0.6307	0.370	0.076		0.408	0.014
35	212.45	786.60	UID	0.2728	0.313	0.049		0.396	0.051

*EPl* ethanolamine plasmalogen, *SEM* standard error of the mean, *RT* retention time.

^a^*m/z*: mass to charge ratio, identified as the molecular ion [M + H] +

^b^Identified molecular species: denoted as the carbon chains at the sn-1 position and fatty acids at the sn-2 position.

^c^Molecular species not identified.

^d^Ratio of the peak area of each EPl molecular species to the total peak area.

^e^The ratio was higher ( >) or lower ( <) in the hypothalami of young brains than in the corresponding of old brains.

Statistical analysis was conducted using two-tailed unpaired *t*-tests. A blue or magenta background indicates a lower or higher ratio, respectively, in old than in young hypothalami.

### EPl biosynthesis enzymes in the hypothalamus

We evaluated mRNA of EPl biosynthesis enzymes in the following regions: The hypothalamus, especially, in the POA, which controls GnRH surge secretion and ovulation; the ARC, which controls GnRH pulsatile secretion and various ovarian functions; the ME, which secretes various hypothalamic hormones into the portal system towards the anterior pituitary^8^. We could not separate the ARC and ME, owing to the very close distance between them.

**Figure 3:**
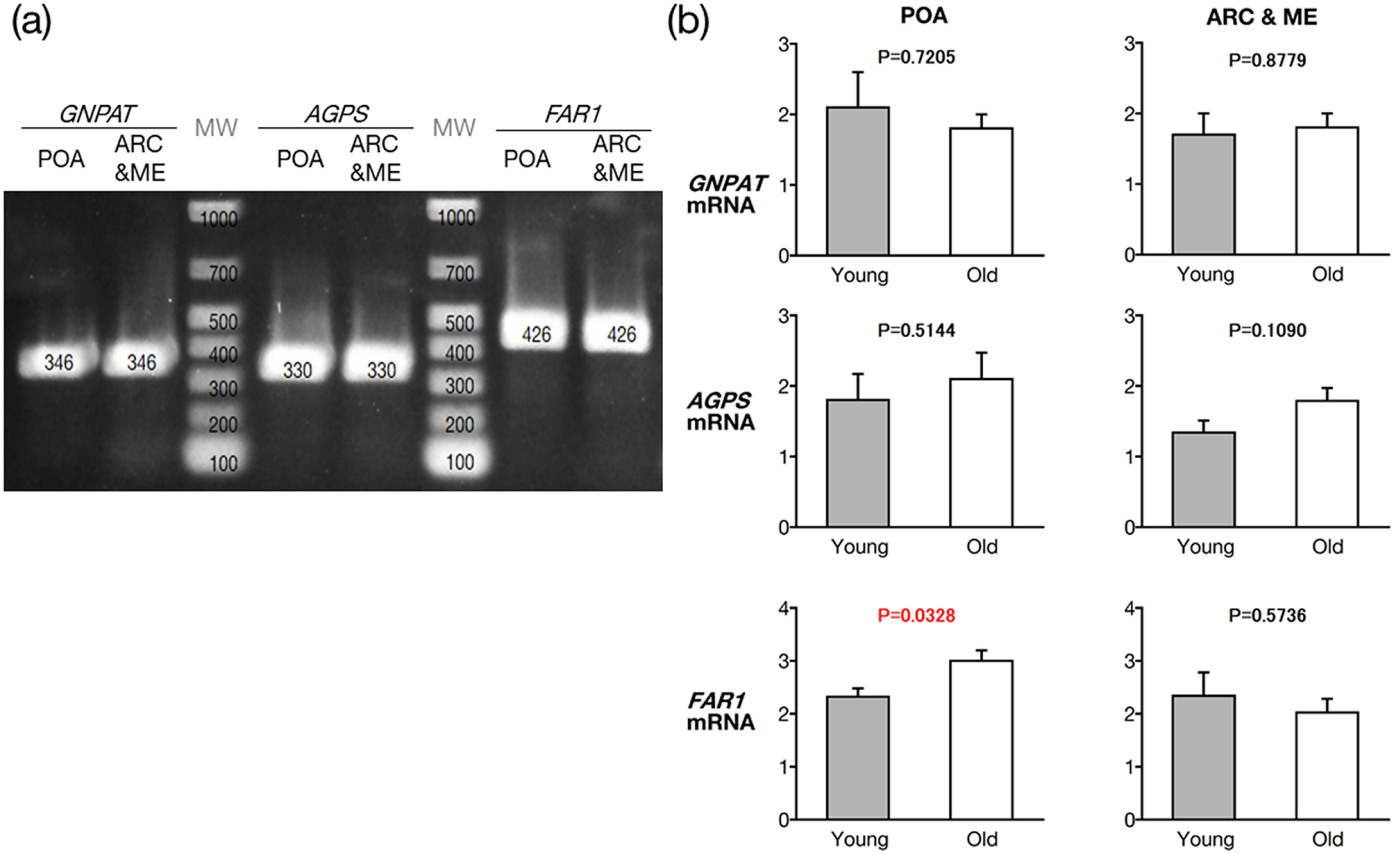
Electrophoresis of amplified DNA produced using RT-PCR to detect expressed mRNA of all three EPl biosynthesis enzymes (i.e. GNPAT, AGPS, and FAR1) (**a**) and compared using quantitative RT-PCRs (**b**). We used primers for each enzyme and cDNA derived from the POA and ARC&ME tissues in fertile, young heifers (26 months old), or old cows (90 months old). (**a**) All lanes were obtained from the same gel; the lanes labelled as enzyme name demonstrate that the obtained DNA products had the expected size (346, 330, and 426 bp, respectively), whereas the other two lanes (MW) correspond to the DNA marker. Two-tailed unpaired *t*-tests was used to determine statistically significant differences in FAR1 in the POA and ARC&ME tissues between the groups. *EPL* ethanolamine plasmalogen, *AGPS* alkylglycerone phosphate synthase, *FAR1* fatty acyl-CoA reductase 1, *POA* preoptic area, *ARC&ME tissues* arcuate nucleus and median eminence-containing intermediate and posterior hypothalamic tissue, *GNPAT* Glyceronephosphate O-acyltransferase, *MW* molecular weight marker.

Reverse transcription-polymerase chain reaction (RT-PCR) detected the mRNA of all three aforementioned EPl biosynthesis enzymes (i.e. *GNPAT*, *AGPS*, and *FAR1*), in the POA and ARC&ME tissues (n = 5, 26 months old; Fig. 3a). Quantitative RT-PCR (RT-qPCR) detected higher *FAR1* levels in the POA, but not in the ARC&ME tissues, of old cows (90.8 ± 1.9 months old; n = 5) than that of fertile young heifers (25.8 ± 0.5 months old; n = 5; Fig. 3b).

### Upregulated GPR61 expression in the anterior pituitary of old cows

**Figure 4:**
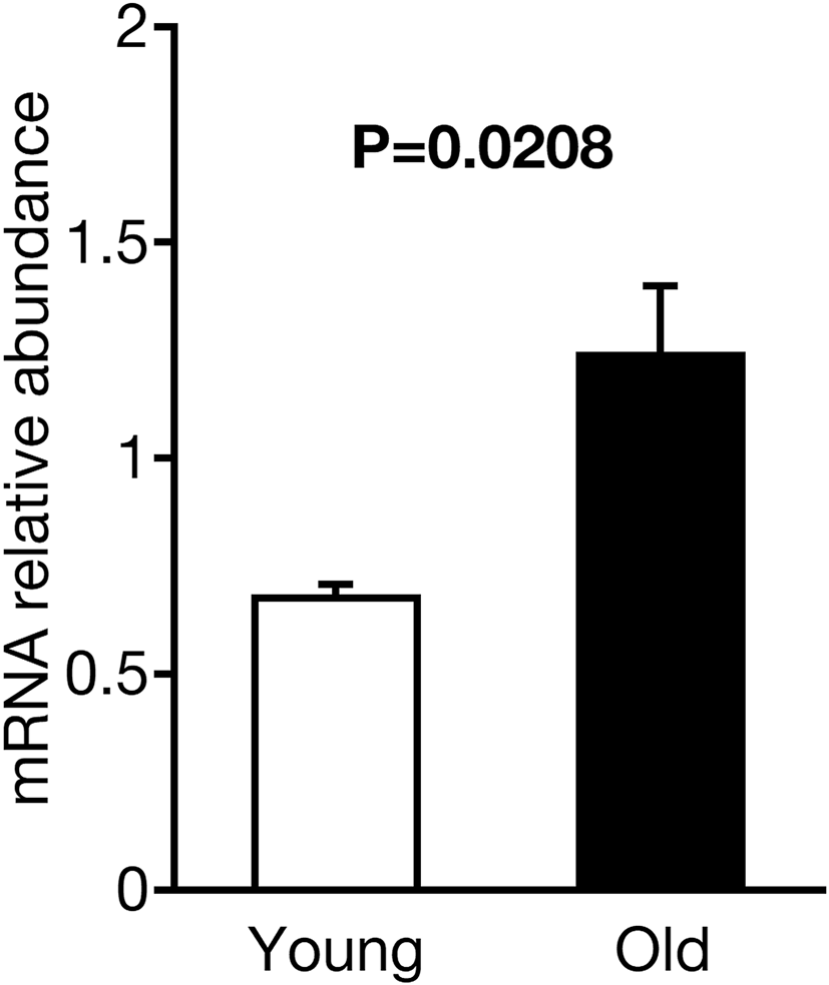
Upregulated G-protein-coupled receptor 61 (GPR61) expression in the anterior pituitary of old cows. Comparison of *GPR61* mRNA expression measured using RT-qPCR. There were significant differences between the groups. Statistical analysis was conducted using two-tailed unpaired *t*-tests.

To estimate GPR61 upregulation in the anterior pituitary induced by decreased hypothalamic EPl in old brains, we used RT-qPCR to compare the mRNA levels of *GPR61* in the anterior pituitary between young heifers (26.2 ± 0.4 months old; n = 5) and old cows (90.2 ± 1.9 months old; n = 5). We observed upregulated (P = 0.0208) *GPR61* expression in the anterior pituitary of old cows compared to that of young heifers (Fig. 4).

## Discussion

**Figure 5:**
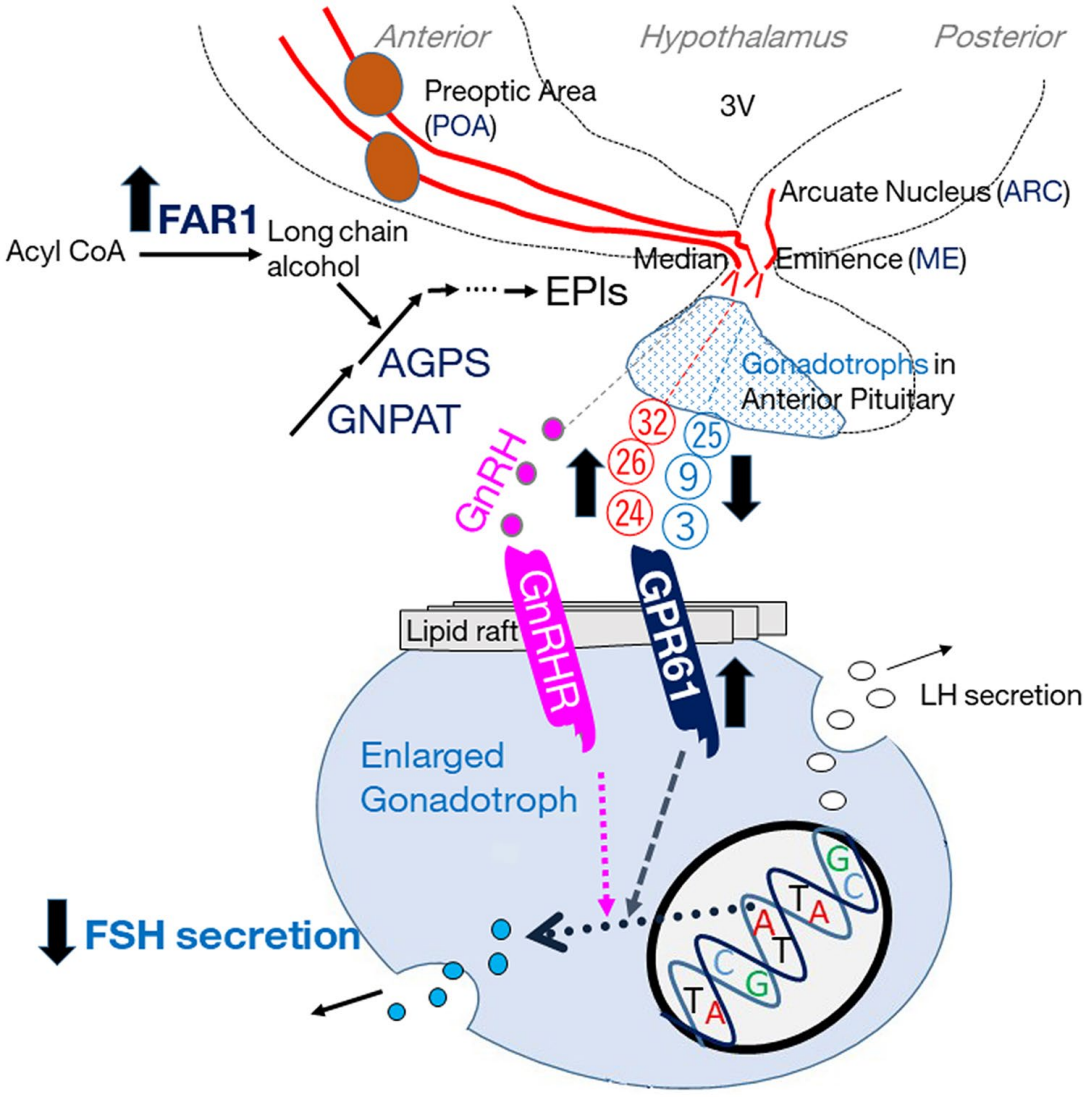
Graphical abstract based on the obtained data and speculation on the relationship between EPl, its biosynthesis enzymes (GNPAT, AGPS, and FAR1) in the anterior hypothalamus containing POA or the posterior hypothalamus containing ARC&ME, and gonadotroph, shown as small dots in the anterior pituitary or as enlarged circle, including the GnRHR, GPR61, and FSH and LH secretions. Red circle numbers (24, 26, and 32) indicate the peak numbers of the EPl molecular species, which were higher in old than young hypothalami. In addition, the blue circle numbers (3, 9, and 25) indicate the peak numbers of the EPl molecular species, which were lower in old than young hypothalami. The up and down bold black arrows indicate the increased or decreased levels of each molecules. We have identified 20 EPl molecular species, demonstrated that the presence of EPl in hypophyseal portal blood was just a speculation, and not evaluated the effect of each EPl molecular species. Finally, EPl produced in other tissues may possibly act on gonadotrophs. *AGPS* alkylglycerone phosphate synthase, *FAR1* fatty acyl-CoA reductase 1, *POA* preoptic area, *ARC&ME tissues* arcuate nucleus and median eminence-containing intermediate and posterior hypothalamic tissue, *GnRHR* gonadotropin-releasing hormone receptor, *FSH* follicle-stimulating hormone, *LH* luteinising hormone.

Our findings suggest that old-brain EPl does not stimulate FSH secretion by pituitary gonadotrophs to the same degree as that of young brains; Fig. 5 illustrates the relationship between EPl, mRNA expression of biosynthesis enzymes in POA, and gonadotroph stimulation, based on the obtained results and speculation. We speculated that EPls may be transported from the hypothalamus to gonadotrophs. Brain EPl stimulates FSH secretion in bovine gonadotrophs, even in the absence of GnRH, through cytoplasmic signalling pathways^4^. The 2D LC–MS system used in this study was merely for analytical purposes and could not be used to separately elute each EPl molecular species; therefore, we did not evaluate the effect of each EPl molecular species. However, our findings suggest that age-based differences in the ratios of EPl molecular species were responsible for reduced gonadotrophic FSH secretion. Interestingly, lysophosphatidylethanolamine, which lacks an acyl group in the sn-2 position, did not affect FSH and LH secretions in our previous study^4^. Therefore, we propose that the acyl group at the sn-2 position is crucial for regulating gonadotrophic secretion. Our findings indicated that C20:4, C22:4, and C18:1 at the sn-2 position may be crucial for stimulating FSH secretion. Moreover, our findings indicated that C20:1 at the sn-2 position play a vital role in inhibiting FSH secretion.

The reaction steps for EPl biosynthesis are as follows: Acylation of dihydroxyacetone phosphate at the sn-1 position by GNPAT, transfer of acyl-DHAP across the enzyme active sites, and finally exchange of the acyl group (fatty acid) for an alkyl group (fatty alcohol generated by FAR1) by AGPS^5^. Therefore, the increased FAR1 levels may partially explain the differences observed in the hypothalamic EPls. However, we could not western blotting for the enzymes because we could not obtain appropriate set of antibodies, and positive and negative controls to perform quantitative assays. Therefore, further studies are warranted to evaluate the changes in enzymes in brains.

Three EPl molecular species were higher in the hypothalamus in old than in young brains. Therefore, an increased suppressive effect of these EPl molecular species is possible. In EPl remodelling, the sn-2 acyl group is removed by a plasmalogen-selective phospholipase A2 and replaced with a different acyl group by lysophospholipid acyltransferases^5^. In an unpublished study using RNA-Seq, we discovered > 25 phospholipase A2 members in the bovine hypothalamus. Therefore, further studies are warranted to clarify the age-related differences in the expression of phospholipase A2 and lysophospholipid acyltransferases in the brain.

Approximately 75% of GPR61-positive cells in the cattle pituitary are gonadotrophs, while the remaining are non-gonadotrophs^2^. Although the precise function of GPR61 remains unclear, GPR61-deficient mice have exhibited hyperphagia-associated obesity^9^. Moreover, GPR61 has been implicated in type 2 diabetes^10^. Therefore, the quantitative and qualitative differences in hypothalamic EPl observed in this study may affect food intake and body weight via GPR61 in non-gonadotroph cells in the anterior pituitary.

Compared with older brains, in this study, younger brains had a lower ratio of glucosylceramide and sphingomyelin peak areas to the total peak area. To the best of our knowledge, there has been no previous report on the relationship between hypothalamic lipids and gonadotrophs, the pituitary, or reproduction. Glucosylceramide may be involved in the central nervous system regulation of body weight and energy homeostasis^11^. Although further studies are warranted, these lipids are unlikely to contribute substantially to the regulation of FSH secretion.

For culturing, we used EPl-rich lipids from the whole brain rather than from the hypothalamus, due to limitations regarding the amount of EPl-rich lipids in the hypothalamus. There was no difference in the first- and second-dimensional HPLC profiles of EPl between the whole brain and the hypothalamus. Therefore, the stimulatory effect of hypothalamic EPl-rich lipids was likely similar to that in the whole brain.

We demonstrated in this study that, in the absence of GnRH, the FSH secretion from anterior pituitary cells stimulated by 0.5 ng/mL young-brain EPl was higher than that stimulated by 50 ng/mL young-brain EPl. We previously reported that excess GnRH (> 1 nM) exhibited a weaker stimulation of LH secretion in the same system of cultured bovine anterior pituitary cells^12^. Therefore, excess EPl may have a weaker stimulatory effect.

Similar to that in women, old age is associated with decreased fertility in cows^7^. The hypothalamus is a brain structure with highly conserved anatomy throughout the vertebrates, owing to its essential function in regulating fundamental aspects of physiological homeostasis and behaviour^13^. Therefore, the results obtained from this study may be generalisable to other species, including humans.

Although the brain contains the highest EPl levels in the body^5^, EPl produced in other tissues may act on the pituitary, which may itself produce EPl to act in an autocrine or paracrine fashion. Therefore, further studies are warranted to evaluate the changes in EPl molecular species in other organs. Especially, whether EPl production in all tissues is modified in the same way by ageing remains to be determined. Specific changes in the brain due to ageing should also be evaluated.

EPl alleviates amyloid-β-induced neurotoxicity, possibly through the vinyl-ether linkage at the sn-1 position^14^, making it a potential therapeutic agent against Alzheimer's disease^15^. Further studies are warranted to determine the therapeutic effect of the molecular species observed in this study on Alzheimer's disease.

Owing to the possible important sex-associated differences, interpreting the obtained data in human females may not be as clear. Old cows have no menopause at the time of slaughter^16,17^. In a recent literature review of 23 selected previous papers^18^, blood FSH concentration in human females approaching menopause decreased in 21 studies, while the remaining two reported an increase. However, it is possible that other factors, besides EPl (e.g. oestradiol and prolactin), may contribute to controlling blood FSH levels in older human females^18^. In conclusion, our findings indicated that age-related qualitative and quantitative differences in brain EPl may be crucially involved in age-related infertility in cows.

## Methods

### Ethics statement

All experiments were performed according to the Guiding Principles for the Care and Use of Animals in the Field of Physiological Sciences (Physiological Society of Japan). All experiments involving animals were approved by the Committee of Yamaguchi University (Approval Number, 301). All cattle were obtained from contract farmers in western Japan. Following the disaster of bovine spongiform encephalopathy in 2002, all cattle born in Japan are registered at birth in a national database, with an individual identification number. Consumers can obtain information regarding the breed, date of birth, farm of origin, and slaughter by querying the server of the National Livestock Breeding Centre of Japan. We verified the above information in this study. All cattle involved in this study were slaughtered for harvesting beef according to the regulation of the Ministry of Agriculture, Forestry, and Fisheries of Japan.

In terms of safety hazards, organic solvents and paraformaldehyde were handled inside a fume hood to prevent its inhalation. Insulating gloves were used when handling acids, liquid nitrogen, and the − 80 °C freezer.

### Brain and anterior pituitary sample collection

We obtained whole brain, hypothalamus, and anterior pituitary samples from healthy, post-pubertal, young Japanese Black heifers and old Japanese Black cows at a local abattoir, following a previously described method^12,19^. After slaughter, the samples were collected, placed in liquid nitrogen within 15 min, and stored at − 80 °C.

We followed previously reported methods^19^ to collect POA and ARC&ME tissue samples from these animals to perform RT-PCR, or RT-qPCR.

All cattle were in the luteal phase, as determined by macroscopic examination of the ovaries and uterus^20^; the anterior pituitary exhibits the highest LH, FSH, GPR61, and GnRH receptor levels in this phase^2,21^. None of the cattle used in the present study were lactating or pregnant and had no follicular cysts, luteal cysts, or other ovarian disorders^22^. The old cows were slaughtered for beef after completing parturition a sufficient number of times, as planned by the farmers.

### Large-scale EPl extraction from whole brains for evaluation with cultured anterior pituitary cells

All organic solvents used in HPLC analysis were of HPLC grade, and purchased from Nacalai Tesque, Inc. (Kyoto, Japan). We used phospholipase A1 (EC 3.1.1.32) from *Aspergillus oryzae* (10,000–13,000 units/g; Mitsubishi Kagaku and Foods Co., Tokyo, Japan). Phospholipase A1 hydrolyses the acyl bond at the sn-1 position of glycerophospholipids; however, it does not act on the alkenyl and alkyl bonds of phospholipids. Therefore, the treatment of total lipids from the brain with phospholipase A1 leaves intact only the ether phospholipids of all classes of glycerophospholipids, including plasmalogens^23^. According to a study reporting plasmalogen extraction^24^, EPl-rich brain lipids were prepared from a mixture of five whole brains for the purposes of cell cultures for 2D LC–MS analyses (details are provided online in the Supplementary Methods).

### Small-scale EPl extraction from hypothalami for 2D LC–MS analysis

Total lipids were extracted from young and old hypothalami using Folch’s method^25^ and treated with phospholipase A1 (details are provided online in the Supplementary Methods).

### 2D LC–MS analysis

We used a novel 2D LC–MS system, as described previously^6^, to analyse the EPl molecular species (details are provided online in the Supplementary Methods).

### Analysis of the effects of EPl-rich lipids on young- or old-brain anterior pituitary cell culture

We obtained anterior pituitaries from healthy, post-pubertal Japanese Black heifers at the local abattoir, using a previously described method^2,4^. The heifers were in the mid-luteal phase. Enzymatic dispersal of anterior pituitary cells was performed using a previously described method^12^, and confirmation of cell viability of > 90% was determined via trypan blue exclusion. Dispersed cells were suspended in Dulbecco’s Modified Eagle’s Medium (DMEM), containing nonessential amino acids (Thermo Fisher Scientific, Waltham, MA, USA), 100 U/mL penicillin, 0.05 mg/mL streptomycin, 10% horse serum, and 2.5% foetal bovine serum. Cells (2.5 × 10^5^ cells/mL, total 0.3 mL) were plated in 48-well culture plates and maintained at 37 °C, in a humidified atmosphere of 5% CO_2_, for 82 h. Each experiment was performed six times with each of the six different pituitary glands, using four wells per treatment. We supplied recombinant human activin A (final concentration, 10 ng/mL; R&D Systems, Minneapolis, MN, USA) to stimulate FSH synthesis 24 h prior to the tests.

To evaluate the effect of young- or old-brain EPl-rich lipids, the initial medium was replaced with 0.25 mL of DMEM containing 0.1% bovine serum albumin and 10 ng/mL activin A, and incubated at 37 °C for 2 h. Treatment was performed by adding 0.5 mL of DMEM alone, or 0.5 mL of DMEM containing various concentrations (final concentrations of 0, 0.05, 0.5, 5, or 50 ng/mL) of young- or old-brain EPl-rich lipids. After incubation at 37 °C for a further 2 h, the medium from each well was collected for radioimmunoassay of LH and FSH concentrations, using a previously reported method^2^. These concentrations were selected based on our previous study^3^.

### RNA extraction, cDNA synthesis, and RT-PCR

Total RNA was extracted using the RNAzol RT isolation reagent (Molecular Research Centre, Inc., Cincinnati, OH, USA), and treated with deoxyribonuclease. The concentration and purity of each RNA sample were evaluated by spectrophotometry (acceptable range, 1.8–2.1) and electrophoresis (28S:18S ratios were 2:1). Complementary DNA was synthesised using the Verso cDNA Synthesis Kit (Thermo Fisher Scientific).

We used previously reported RT-PCR methods^2^ to detect mRNAs of *GNPAT* (NCBI reference sequence, NM_001103286), *AGPS* (NCBI reference sequence, NM_001206719), and *FAR1* (NCBI reference sequence, NM_001099032) in the POA (n = 5) and ARC&ME (n = 5) tissues. Details of primers are provided online in Supplementary Table S5.

We used previously reported RT-qPCR methods^26^ to compare the mRNA expressions of *GNPAT*, *AGPS*, and *FAR1* in young and old POA and ARC&ME tissues using specific primers (details of primers are provided online in Supplementary Table S6), the CFX96 real-time PCR System (Bio-Rad, Hercules, CA, USA), Power SYBR Green PCR Master Mix (Thermo Fisher Scientific), a six-point relative standard curve, no-template control, and no reverse transcription control. The expression of each enzyme was normalised against the geometric mean of the expression of two house-keeping genes, *tyrosine 3-monooxygenase/tryptophan 5-monooxygenase activation protein zeta* (*YWHAZ*; NCBI reference sequence, NM_174814.2) and *succinate dehydrogenase complex flavoprotein subunit A* (*SDHA*; NCBI reference sequence, NM_174178.2). The two housekeeping genes were reported for ewe’s hypothalamus^27^ and presented 100% homology with the respective bovine genes.

Additionally, we used previously reported RT-qPCR methods^26^ to compare mRNA expression of *GPR61* (NCBI reference sequence, NM_001038571) in young and old anterior pituitaries using the reported. The expression of *GPR61* was normalised against the geometric mean of the expression of two housekeeping genes, *glyceraldehyde-3-phosphate dehydrogenase* (*GAPDH*; NCBI reference sequence, NM_001034034) and *RAN binding protein 10* (*RANBP10*; NCBI reference sequence, NM_001098125).

### Statistical analysis

Data were analysed using StatView version 5.0 for Windows (SAS Institute, Inc., Cary, NC, USA). The Shapiro–Wilk or the Lilliefors test was used to evaluate the normality or log-normality of each variable, respectively—all variables were normally distributed. We used Via Grubb’s test and verified that there were no outliers for any of the variables. Differences in LH or FSH concentrations were analysed using one-factor analysis of variance, with *post-hoc* comparisons performed using Fisher’s protected least significant difference test. We compared the measured values obtained from young and old brains using two-tailed unpaired *t*-tests. The level of significance was set at *P* < 0.05. Data are expressed as means ± standard errors of the mean.

## Supplementary Information

Supplementary Information

## Data Availability

The datasets of the present study are available from the corresponding authors upon reasonable request.
