# Supplementary material for: Reduced gonadotroph stimulation by ethanolamine plasmalogens in old bovine brains
Source: Sci Rep. 2021 Feb 26;11:4757. doi: 10.1038/s41598-021-84306-6 (PMC7910589; doi:10.1038/s41598-021-84306-6)
Supplement: Supplementary file 1 — Supplementary Information [file 41598_2021_84306_MOESM1_ESM.pdf]

Reduced gonadotroph stimulation by ethanolamine plasmalogens in old bovine brains

Hiroya Kadokawa, Miyako Kotaniguchi, Onalenna Kereilwe, Shinichi Kitamura

Contents of this Supplementary Material

Supplementary Methods

Page 3-5: Large-scale EPl extraction from whole brains for evaluation with cultured anterior pituitary cells.

Page 6-7: Small-scale EPl extraction from the hypothalamus for 2D LC-MS analysis

Page 8-10: 2D LC-MS analysis

Supplementary Tables

Page 11. Supplementary Table S1. Details of P-values presented in Figure 1a.

Page 12. Supplementary Table S2. Details of P-values presented in Figure 1b.

Page13. Supplementary Table S3. Details of P-values presented in Figure 1c.

Page 14. Supplementary Table S4. Details of P-values presented in Figure 1d.

Page 15. Supplementary Table S5. Primers used in this study for RT-PCR.

Page 16. Supplementary Table S6. Primers used in this study for quantitative RT-PCR.

Supplementary methods

Large-scale EPl extraction from whole brains for evaluation with cultured anterior pituitary cells

Five young or old whole brains were minced in a food processor (DLC-NXJ2PS, Conair Japan G. K., Tokyo, Japan), pooled, frozen at −80°C, and vacuum-dried (ADP200, Yamato Scientific Co. Ltd., Tokyo, Japan). Vacuum-dried brain tissue was extracted by incubation in ethanol (brain tissue/ethanol, 1:10, v/v) at 40 °C for 8 h with shaking. After centrifugation at 10,800 × *g* for 1 hour at 25 °C, the supernatant was collected and dried using a rotary evaporator (N2110; Tokyo Rikakikai Co. LTD., Tokyo, Japan). The remaining lipids were collected in several 50-mL glass centrifuge tubes and dissolved in diluted acetone (acetone/water, 2:1, v/v; lipids/diluted acetone, 1:10, v/v). After storing at 4 °C for 1 hour, the solutions were centrifuged at 1,200 × *g* for 20 min at 4 °C, and the supernatant was subsequently removed. The remaining precipitates were dissolved in diluted acetone (acetone/water, 1:1 v/v; precipitate/diluted acetone, 1:10 v/v). The solutions were centrifuged at 1,200 × *g* for 20 min at 4 °C to collect the precipitates, which were subsequently mixed with cold acetone (precipitate/acetone, 1:10 v/v), and stored at –20 °C overnight. Thereafter, the acetone-treated precipitates were centrifuged

at 1,200 × *g* for 20 min at 4 °C and the supernatant was removed. The remaining precipitates were dissolved in a hexane and acetone mixture (hexane/acetone, 7:3 v/v; precipitate/mixture, 1:10 v/v). This solution was subjected to centrifugation at 1,200 × *g* for 30 min at 4 °C, and the supernatant was collected into a flask and dried using a rotary evaporator. After evaporation, the remaining lipids were treated with 20 mg/mL phospholipase A1 dissolved in 0.1 M citric acid buffer (pH 4.5) at 50 °C, in a volume ratio of 1:10, under a low oxygen atmosphere (air pressure, 50 kPa), in a rotary evaporator flask for 2 h. Subsequently, the enzyme-treated sample was mixed with a 1:1 (v/v) mixture of hexane and acetone (sample/mixture, 1:6 v/v) and transferred to a separating funnel for collection of the upper layer. This extraction was repeated two more times. The upper layer was transferred to a flask for drying using a rotary evaporator. After evaporation, the residual lipids were dissolved in acetone (lipids/acetone, 1:10 v/v) in glass tubes, and stored at -20 °C overnight. Subsequently, the solutions were centrifuged at 1,200 × *g* for 20 min at 4 °C to collect the precipitates, which were washed with acetone (precipitate/acetone, 1:10 v/v), and recentrifuged at 1,200 × *g* for 20 min at 4 °C. The resulting precipitates were dissolved in a hexane/acetone mixture (hexane/acetone, 7:3 v/v; precipitate/mixture, 1:10 v/v). The solutions were centrifuged at 1,200 × *g* for 20 min at 4 °C and the supernatant was

collected for evaporation. The remaining lipids after evaporation were dissolved in a mixture of hexane, acetone, and water (hexane/acetone/water, 3:3:1 v/v; lipids/mixture, 1:10 v/v), and transferred to a separating funnel. After shaking the separating funnel, the upper layer was collected. This extraction was repeated two more times. The combined upper layer extracts were evaporated using a rotary evaporator to obtain EPl-rich lipids. Aliquots of these lipids were vacuum-packed and stored at –30 °C prior to analysis.

Small-scale EPl extraction from the hypothalamus for 2D LC-MS analysis

All organic solvents were prepared to a 0.01% (w/v) concentration in 2,6-di*tert*-butyl-4-methylphenol. Briefly, frozen hypothalamus samples were thawed and homogenised in twice the volume of methanol, and stored overnight at – 20 °C. The homogenate was subsequently vortexed for re-suspension. Next, 1 mL of the homogenate was transferred to a 10-mL glass centrifuge tube, mixed with 2 mL of methanol and 6 mL of chloroform, and vortexed at room temperature for 10 min. It was subsequently centrifuged at 1,200 × *g* for 15 min at 25 °C. The upper layer was collected in a glass tube for heat drying (45 °C) under a gentle stream of N_2 gas. This extraction was repeated thrice for each sample. After drying, 3 mL of chloroform/methanol (2:1, v/v) was added into the tube to dissolve the residue. This mixture was centrifuged at 1,200 × g for 15 min at 25 °C, and the supernatant was collected into another 10 mL glass centrifuge tube for heat drying (45 °C) under a N_2 gas stream. This extraction was repeated two more times for each sample. After drying, 1.5 mL of 20 mg/mL phospholipase A1 (dissolved in 0.1 M citric acid buffer, pH 4.5) was added to the tube, which was filled with N_2 gas before capping, and the sample was incubated at 45 °C for 2 h. When the sample became milky, 10 mL of acetone/hexane (2:1 v/v) was added. The sample was vortexed and centrifuged at 1,200 × *g* for 15 min

at 25 °C to collect the upper layer into another glass centrifuge tube for heat drying (45 °C) under a N_2 gas stream. This extraction was repeated two more times for each sample. After drying, 10 mL of cold acetone was added, and the sample was vortexed and incubated overnight at –20 °C. Thereafter, the sample was centrifuged at 1,200 × *g* for 15 min at 25 °C to remove the supernatant. This washing step was repeated two more times. After the supernatant was removed, the remaining precipitate was dissolved in 10 mL of hexane/acetone (7:3 v/v), and centrifuged at 1,200 × *g* for 15 min at 25 °C to collect the supernatant into a glass tube for heat drying (45 °C) under a N_2 gas stream. After drying, 6 mL of a hexane/acetone mixture (7:3 v/v) was added, the sample was vortexed, and 0.9 mL of water was added. After shaking, the sample was centrifuged at 1,200 × *g* for 15 min at 25 °C to collect the upper layer into a glass tube of pre-determined weight. This extraction was repeated two more times. After heat drying (45 °C) under a N_2 gas stream, the tube was weighed to calculate the weight of the obtained lipids. The tube was filled with N_2 gas, capped, vacuum-packed, and transported on dry ice to the 2D LC-MS system.

2D LC-MS analysis

This system, as previously reported[6], consists of: (1) normal-phase HPLC, to separate targeted phospholipids (phosphatidylethanolamines, in this study) from other lipid classes, in the first column (first-dimensional HPLC separation); (2) a switching valve, trapping column, and make-up pump, to trap the target lipid class; (3) reverse-phase HPLC to separate the target lipid classes, in the second column (second-dimensional HPLC separation); (4) a charged aerosol detector and electrospray ionization-mass spectrometer to identify and quantify EPl molecular species. The components of the 2D LC-MS system, such as the columns, pumps, autosampler, detectors, electrospray ionization-mass spectrometer, and the software, were the same as those previously reported[6]. First-dimensional separation was performed using a YMC-Pack PVA-Sil [250 mm length (L) × 4.6 mm internal diameter (I.D.), 5-µm column; YMC Co. Ltd., Kyoto, Japan]. The HPLC separation temperature and flow rate were set to 30 °C and 1.0 mL/min, respectively. The lipid sample was prepared at a concentration of 5 mg/mL in chloroform/methanol (2:1 v/v), and a 0.02-mL aliquot was injected into the 2D-HPLC system. The mobile phases, A, B, and C, were hexane, 2-methoxy-2-methylpropane, and methanol, respectively. The solvent gradient program was as follows: 0–7 min A/B/C (v/v/v%) 88/10/2; 7–12 min A/B/C (v/v/v%) 2/88/10;

12–22 min A/B/C (v/v/v%) 2/28/70; 22–32 min A/B/C (v/v/v%) 2/28/70; 32–35 min A/B/C (v/v/v%) 88/10/2. The separation profile was monitored at 210 nm using a variable-wavelength detector. The trapping column was conditioned with a make-up solvent (water/acetonitrile, 40/60, v/v%) before measurement, and the flow rate of the make-up pump was set to 5.0 mL/min. The temperature of the trapping column was set to 27 °C. When the targeted phospholipid fraction was eluted from the first column, the switching valve was changed to mix the targeted phospholipid fraction with the make-up solvent and trap it in a high-carbon-content octadecyl-silica column (YMC-Pack Pro C18 RS; carbon content: 22%; 30 mm L × 4.6 mm I.D., 5-μm column; YMC Co. Ltd.). Thereafter, the flow channel was switched to the second-dimensional HPLC component. EPl molecular species were separated on a hybrid silica-based column (YMC Triart-C18; 250 mm L × 4.6 mm I.D., 3-μm column; YMC Co. Ltd.) at 40 °C. The mobile phase was acetonitrile/methanol/20 mM ammonium acetate (25/68.5/6.5, v/v/v%) at a flow rate of 1 mL/min. The eluent from the second column was split into a charged aerosol detector and electrospray ionization-mass spectrometer. The acquisition range and N_2 gas pressure of the charged aerosol detector were 500 pA and 241.3 kPa, respectively. An electrospray ionization-mass spectrometer was used to identify the species (positive ion mode; N_2 sheath gas flow rate: 5 units; capillary temperature:

250 °C; source voltage: 5 kV; capillary voltage: 30 V; tube lens voltage: 80 V). The data-dependent mode was set up with two scan events: One to collect the full mass spectrum of all the ions in the sample (MS range *m/z*: 300–2,000), and the other to collect the tandem MS (MS^2) spectra of the most intense ions at each time point from the MS spectrum in the scan event. The dynamic exclusion setting was as follows: The repeat count for each ion was set to 3, with a report duration of 10 seconds, an exclusion list size of 30, and an exclusion duration of 30 seconds. Collision-induced dissociation was conducted with an isolation width of 4 Da and a normalised collision energy of 35. Each sample was analysed in duplicate, and each relative standard deviation of the retention time and peak area was less than 0.05% and 0.93%, respectively.

Supplementary Table S1. Details of P-values presented in Figure 1a

	Control	GnRH	0.05	0.5	5	50
Control	-	0.0001	0.0174	0.0001	0.0001	0.0005
GnRH	0.0001	-	0.0001	0.153	0.0745	0.0001
0.05	0.0174	0.0001	-	0.0001	0.0001	0.175
0.5	0.0001	0.153	0.0001	-	0.7054	0.0002
5	0.0001	0.0745	0.0001	0.7054	-	0.0007
50	0.0005	0.0001	0.175	0.0002	0.0007	-

The P-values were calculated using Fisher's protected least significant difference test following one-factor analysis of variance to compare the effects of various concentrations (ng/mL) of young bovine brain EPl in media lacking GnRH on FSH secretion from cultured anterior pituitary cells.

EP1, ethanolamine plasmalogen; FSH, follicle-stimulating hormone; GnRH, gonadotropin-releasing hormone.

Supplementary Table S2. Details of P-values presented in Figure 1b

	Control	GnRH	0.05	0.5	5	50
Control	-	0.0001	0.4791	0.0814	0.0756	0.0653
GnRH	0.0001	-	0.0001	0.0001	0.0001	0.0001
0.05	0.4791	0.0001	-	0.2859	0.2699	0.2409
0.5	0.0814	0.0001	0.2859	-	0.9702	0.9132
5	0.0756	0.0001	0.2699	0.9702	-	0.9428
50	0.0653	0.0001	0.2409	0.9132	0.9428	-

The P-values were calculated using Fisher's protected least significant difference test following one-factor analysis of variance to compare the effects of various concentrations (ng/mL) of old bovine brain EPl in media lacking GnRH on FSH secretion from cultured anterior pituitary cells.

EP1, ethanolamine plasmalogen; FSH, follicle-stimulating hormone; GnRH, gonadotropin-releasing hormone.

Supplementary Table S3. Details of P-values presented in Figure 1c

	Control	GnRH	0.05	0.5	5	50
Control	-	0.0385	0.4119	0.1774	0.1222	0.4276
GnRH	0.0385	-	0.1928	0.4398	0.5705	0.1839
0.05	0.4119	0.1928	-	0.5869	0.454	0.978
0.5	0.1774	0.4398	0.5869	-	0.8356	0.5682
5	0.1222	0.5705	0.454	0.8356	-	0.4378
50	0.4276	0.1839	0.978	0.5682	0.4378	-

The P-values were calculated using Fisher's protected least significant difference test following one-factor analysis of variance to compare the effects of various concentrations (ng/mL) of young bovine brain EPl in media lacking GnRH on LH secretion from cultured anterior pituitary cells.

EP1, ethanolamine plasmalogen; GnRH, gonadotropin-releasing hormone; LH, luteinising hormone.

Supplementary Table S4. Details of P-values presented in Figure 1d

	Control	GnRH	0.05	0.5	5	50
Control	-	0.0343	0.2757	0.9753	0.8383	0.7713
GnRH	0.0343	-	0.0023	0.032	0.0533	0.0639
0.05	0.2757	0.0023	-	0.2892	0.1981	0.1707
0.5	0.9753	0.032	0.2892	-	0.8142	0.7477
5	0.8383	0.0533	0.1981	0.8142	-	0.9309
50	0.7713	0.0639	0.1707	0.7477	0.9309	-

The P-values were calculated using Fisher's protected least significant difference test following one-factor analysis of variance to compare effects of various concentrations (ng/mL) of old bovine brain EPl in media lacking GnRH on LH secretion from cultured anterior pituitary cells.

EP1, ethanolamine plasmalogen; GnRH, gonadotropin-releasing hormone; LH, luteinising hormone.

Supplementary Table S5. Primers used in this study for RT-PCR

Gene		Primer sequence 5'-3'	Position		Size
			Nucleotide	Exon	(bp)
GNPAT	F	GAACCGGCCATCCATAGACT	1,341–1,360	9	346
	R	GACATCCTCTTTCCTGAACCCA	1,665–1,686	11–12	
AGPS	F	TTCAGCACTGTGGGAGGATG	929–948	9	330
	R	TGCAGGAGCACATCTCTGTTT	1,238–1,258	11–12	
FAR1	F	AGCCTCTGCGACTAGGATCA	37–56	1–2	426
	R	AGCTGTCGTGTAGCAATCACA	442–462	4	
GPR61	F	CATCAACGTGGAGCGCTACTAT	1,130–1,151	3	62
	R	GCGTCATTCGCACCTCATAA	1,172–1,191	3	

These primers were designed using Primer3 (https://www.ncbi.nlm.nih.gov/tools/primer-blast/).

AGPS, alkylglycerone phosphate synthase; FAR1, fatty acyl-CoA reductase 1; GNPAT, glyceronephosphate O-acyltransferase; GPR61, G-protein-coupled receptor 61; RT-PCR, reverse transcription-polymerase chain reaction.

Supplementary Table S6. Primers used in this study for quantitative RT-PCR

Gene	Primer sequence 5'-3'		Position		Size
			Nucleotide	Exon	(bp)
GNPAT	F	GAACCGGCCATCCATAGACT	1,341–1,360	9	109
	R	AGCAGGTTCATTATCAGGCCA	1,429–1,449	9–10	
AGPS	F	TCCTTGGGACAGGGTTGTAGA	1,606–1,626	16–17	126
	R	CCTGCGTCGTAAGTCTGTGT	1,712–1,731	18	
FAR1	F	TTGTTGGTGCCAGTTGGAAA	750–769	5	135
	R	CAAGATCTGCAAGGGCATTGT	864–884	7	
YWHAZ	F	AGACGGAAGGTGCTGAGAAA	256–275	2	123
	R	CGTTGGGGATCAAGAACTTT	359–378	3	
SDHA	F	CATCCACTACATGACGGAGCA	428–448	5	90
	R	ATCTTGCCATCTTCAGTTCTGCTA	494–517	5	
GAPDH	F	TGGTGAAGGTCGGAGTGAAC	73–92	2	91
	R	ATGGCGACGATGTCCACTTT	144–163	3	
RANBP10	F	CCCAGTCCTACCAGCCTACT	4,436–4,455	14	133
	R	CCCCCAGAGTTGAATGACCC	4,549–4,568	14	
GPR61	F	CATCAACGTGGAGCGCTACTAT	1,130–1,151	3	62

R	GCGTCATTCGCACCTCATAA	1,172–1,191	3

The primers of *GNPAT*, *AGPS,* and *FAR1* were designed using Primer3, whereas those of *YWHAZ* and *SDHA* were reported for the ewe's hypothalamus[31], and presented a 100% homology with the respective bovine genes.

AGPS, alkylglycerone phosphate synthase; FAR1, fatty acyl-CoA reductase 1; GAPDH, Glyceraldehyde 3-phosphate dehydrogenase; GNPAT, glyceronephosphate O-acyltransferase; GPR61, G-protein-coupled receptor 61; RT-PCR, reverse transcription-polymerase chain reaction; SDHA, Succinate dehydrogenase complex flavoprotein subunit A; YWHAZ, Tyrosine 3-Monooxygenase/Tryptophan 5-Monooxygenase Activation Protein Zeta.
